# Intestinal epithelial cell-derived components regulate transcriptome of *Lactobacillus rhamnosus* GG

**DOI:** 10.3389/fmicb.2022.1051310

**Published:** 2023-01-04

**Authors:** Kasey Schalich, Seesandra Rajagopala, Suman Das, Ryan O’Connell, Fang Yan

**Affiliations:** ^1^Department of Pediatrics, Vanderbilt University Medical Center, Nashville, TN, United States; ^2^Department of Medicine, Vanderbilt University Medical Center, Nashville, TN, United States; ^3^Department of Pathology, Microbiology and Immunology, Vanderbilt University Medical Center, Nashville, TN, United States; ^4^Department of Pathology, University of Utah, Salt Lake City, UT, United States; ^5^Department of Cell and Developmental Biology, Vanderbilt University, Nashville, TN, United States

**Keywords:** bacterial transporter, bacterial metabolism, commensal bacterium, intestinal epithelial cell, RNA sequencing, the microbial-host interaction, transcriptome

## Abstract

**Introduction:**

Intestinal epithelial cells (IECs) provide the frontline responses to the gut microbiota for maintaining intestinal homeostasis. Our previous work revealed that IEC-derived components promote the beneficial effects of a commensal and probiotic bacterium, *Lactobacillus rhamnosus* GG (LGG). This study aimed to elucidate the regulatory effects of IEC-derived components on LGG at the molecular level.

**Methods:**

Differential gene expression in LGG cultured with IEC-derived components at the timepoint between the exponential and stationary phase was studied by RNA sequencing and functional analysis.

**Results:**

The transcriptomic profile of LGG cultured with IEC-derived components was significantly different from that of control LGG, with 231 genes were significantly upregulated and 235 genes significantly down regulated (FDR <0.05). The Clusters of Orthologous Groups (COGs) and Gene Ontology (GO) analysis demonstrated that the predominant genes enriched by IEC-derived components are involved in nutrient acquisition, including transporters for amino acids, metals, and sugars, biosynthesis of amino acids, and in the biosynthesis of cell membrane and cell wall, including biosynthesis of fatty acid and lipoteichoic acid. In addition, genes associated with cell division and translation are upregulated by IEC-derived components. The outcome of the increased transcription of these genes is supported by the result that IEC-derived components significantly promoted LGG growth. The main repressed genes are associated with the metabolism of amino acids, purines, carbohydrates, glycerophospholipid, and transcription, which may reflect regulation of metabolic mechanisms in response to the availability of nutrients in bacteria.

**Discussion:**

These results provide mechanistic insight into the interactions between the gut microbiota and the host.

## Introduction

The community of microbes in humans exhibits interindividual differences in composition while sharing functional core genes and metabolic modules. The gut microbiota and the host establish mutualistic relationships that play important roles for supporting human health and providing a nutrient-enriched environment for the microbiota (Human Microbiome Project Consortium, 2012; Lloyd-Price et al., 2016). In addition to nutrient absorption and mucosal immune defense (Allaire et al., 2018), the monolayer of intestinal epithelial cells (IECs) serves as the initial interface to the community of the gut microbiota. Commensal bacteria promote intestinal development and regulate the intestinal epithelial functions by preserving the epithelial barrier integrity, promoting cell survival, stimulating the production of antibacterial substances and cell-protective proteins, enhancing protective immune responses, and inhibiting proinflammatory cytokine production (Thomas and Versalovic, 2010; Yan and Polk, 2020). Research has revealed the mechanisms underlying the action of commensal bacteria to be through commensal bacterium-derived metabolites and products. Bacteria can metabolize dietary fiber to produce short-chain fatty acids (SCFAs) to regulate mucosal defense in the host (Bilotta and Cong, 2019). For example, the SCFA butyrate can reinforce the epithelial barrier and enhance wound healing through promoting the expression of tight junction proteins (Wang et al., 2020). Furthermore, commensal bacterium-derived protein products have shown beneficial effects on IECs (Yan and Polk, 2020). A soluble protein derived from probiotic bacteria has been demonstrated to activate EGF receptor and its downstream target, Akt, in IECs to stimulate protective cellular responses against intestinal inflammation (Yan et al., 2011, 2013; Wang et al., 2014, 2017; Yoda et al., 2014). Thus, the interactions between IECs and the gut microbiota contribute to maintaining intestinal homeostasis.

The influence of the host on the gut microbiota is an active area of research. The composition of the gut microbiome is impacted by host factors including genetics, immunological responses, and diseases, as well as environmental factors such as diet and nutrition (Spor et al., 2011; Donaldson et al., 2016). Accumulating evidence has begun to reveal the mechanisms by which the host actively participates in regulating bacterial adaptation, growth, and function, indicating that IECs may play significant regulatory roles. Studies have revealed that MHC class II molecules in IECs participate in regulation of the microbiota composition because the lack of MHC class II in IECs results in a decrease in microbial-bound IgA, regulatory T cells and immune repertoire selection, which is associated with the increase in interindividual microbiota variation and altered proportions of two taxa in the ileum (Stephens et al., 2021). Current findings strongly suggest that molecular investigation into the influence of host factors on the microbiome is needed to further understanding of the microbial-host mutualistic interactions.

*Lactobacillus rhamnosus* GG (LGG) is a well-studied commensal and probiotic bacterium (Capurso, 2019). Clinical evidence suggests multiple health benefits conferred by LGG, such as LGG mitigated gastroenteritis (Szajewska et al., 2019), antibiotic associated diarrhea (Szajewska and Kolodziej, 2015), and asthma and atopic disorders in children (Uwaezuoke et al., 2022). However, it has been challenging for prior clinical trials to assess the bioavailability and biopharmacology of probiotics, including LGG in the gastrointestinal tract. To overcome these concerns, understanding the mechanisms of probiotic action and the potential effects of the host on probiotic effectiveness is crucial to modulate the efficacy of probiotics on human health. Animal studies and *in vitro* assays have demonstrated beneficial effects of LGG on intestinal integrity and mucosal immune homeostasis (Segers and Lebeer, 2014). For example, LGG has been shown to block cytokine-induced apoptosis(Yan and Polk, 2002) and promote expression of cytoprotective heat shock proteins in IECs (Tao et al., 2006). We previously used LGG as a commensal and probiotic bacterial model to demonstrate that IEC-derived components promote the protective effects of LGG on IECs (Yang et al., 2019). To deepen our understanding of functional regulation of microbes by host, we aimed to identify novel effects of IEC-derived components on LGG by investigating the impact of IECs on LGG at the molecular level through functional analysis of the transcriptomic effects of IEC-derived components on LGG. We identified differentially expressed genes involved in enabling nutrient acquisition and other cellular responses which are necessary for bacterial growth. We further confirmed that IEC-derived components promoted LGG growth *in vitro*. Genes encoding enzymes involved in metabolism in LGG cultured with IEC-derived components were downregulated, which reflects nutrition repression in that enzymes for metabolism are repressed in the presence of sufficient nutrients. These results provide a proof-of-concept model to define a novel mechanism underlying microbial-host interactions.

## Results

### IEC-derived components regulate the transcriptomic profile of LGG

In this study, we investigated if IEC-derived components exerted regulatory effects on LGG at the gene transcription level. A colonic epithelial cell line, young adult mouse colonic (YAMC) epithelial cells, were cultured in fetal bovine serum-free RPMI-1640 medium for 24 h to generate conditioned medium (CM). LGG was cultured with YAMC-CM or RPMI-1640 medium as a control for 24 h at 37°C, then LGG RNA was isolated for RNA sequencing. We examined the transcriptomic profile of LGG at 24 h-culture with YAMC-CM because LGG reached the late exponential phase and entered the early stationary phase under this culture condition. The genes for bacterial growth and survival are highly expressed in the exponential phase and the stationary phase, respectively (Jaishankar and Srivastava, 2017). Thus, it is possible that identifying molecular regulatory signatures of YAMC-CM at the timepoint between the exponential and the stationary phase could underpin the diverse benefits of YAMC-CM for LGG.

**Figure 1:**
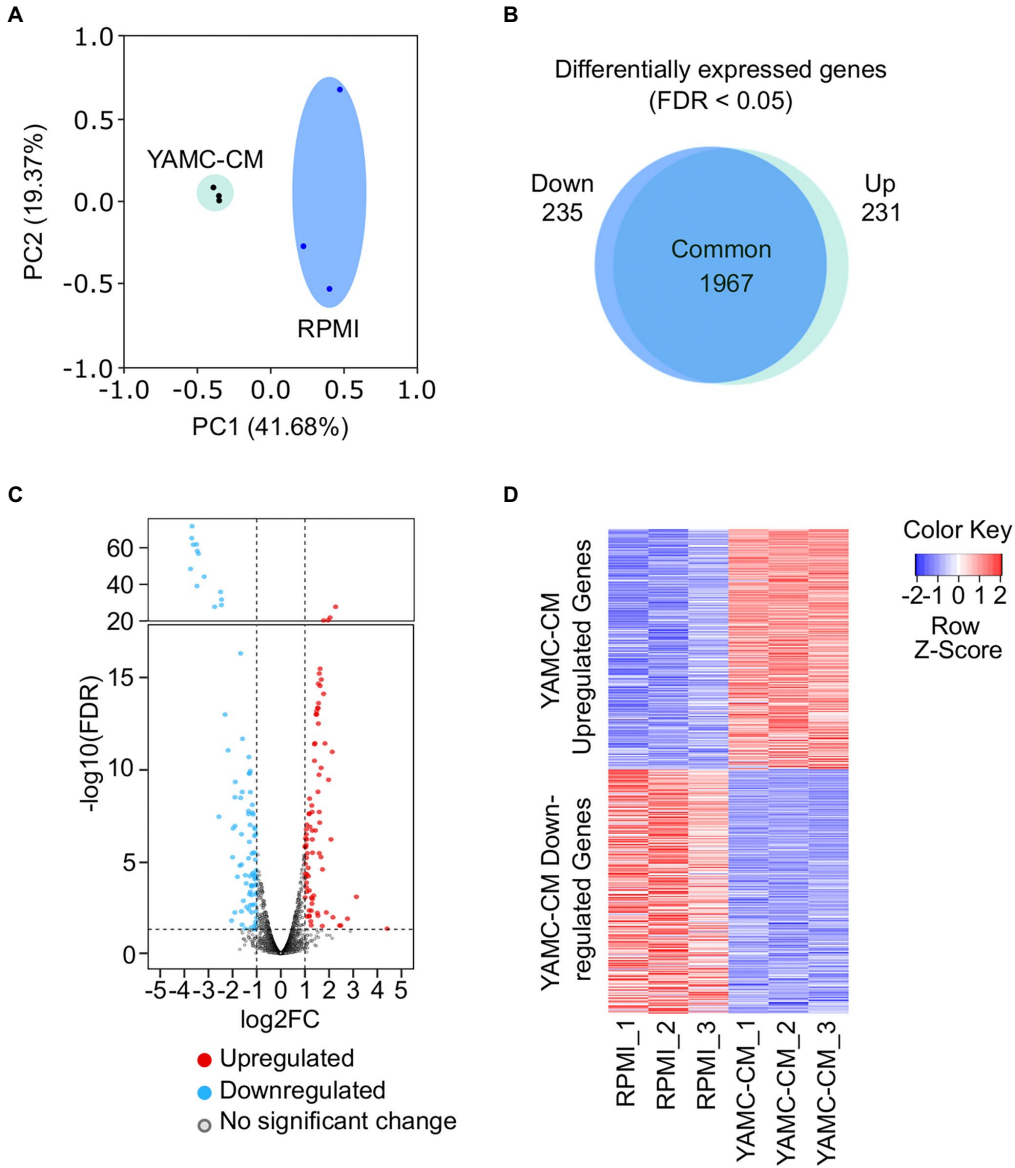
RNA sequencing of LGG identifies differentially expressed genes induced by YAMC conditioned medium (YAMC-CM). **(A)** PCA plot depicting the YAMC-CM treated LGG group and the RPMI treated LGG group (*n* = 3/group). **(B)** Scaled venn diagram demonstrating the nearly equal number of genes upregulated and downregulated (FDR < 0.05) in both study groups. **(C)** Volcano plot illustrating those genes upregulated (red) and downregulated (blue) after YAMC-CM treatment, with thresholds set at log_2_ (FC) of −1 and 1 (*x*-axis) and -log_10_ (FDR) = 0.05 (*y*-axis). **(D)** Heatmap depicting the statistically significant (FDR < 0.05) YAMC-CM upregulated and downregulated genes.

Principal component analysis (PCA) indicates that the transcriptional profile of LGG treated with YAMC-CM is significantly distinct from that of control LGG (Figure 1A). Differential expression analysis identified 231 genes were significantly upregulated and 235 genes were significantly down regulated (FDR < 0.05) in LGG treated with YAMC-CM compared to RPMI (Figure 1B). The log_2_FC of these upregulated (red) and down-regulated (blue) genes are displayed in the volcano plot (Figure 1C). The heatmap clearly illustrates the magnitude difference of the differentially expressed genes of LGG between YAMC-CM and RPMI groups (Figure 1D).

**Figure 2:**
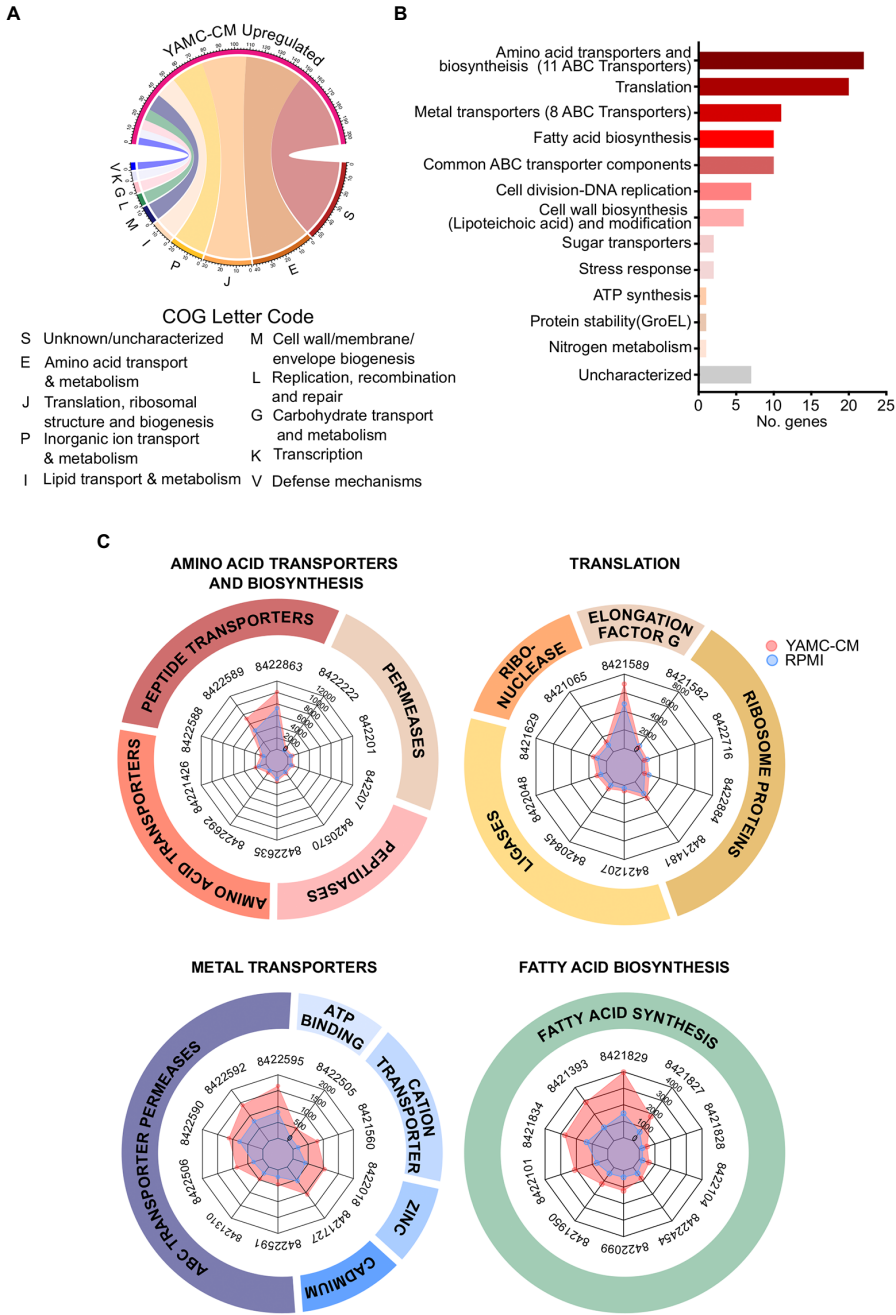
Identification of 231 differentially upregulated LGG genes by YAMC conditioned medium (YAMC-CM). **(A)** The 231 upregulated genes (FDR < 0.05) were classified by the COG system. **(B)** Sorting of the 231 upregulated genes by abundance differential with the control RPMI group identified the top 100 YAMC-CM upregulated genes by expression difference, which were categorized according to GO biological process and molecular function to identify the number of genes associated with specific biological functions. **(C)** Radar plots comparing the YAMC-CM and RPMI mean group expression values (in CPM) for the top 10 most abundant genes within the top four enriched categories amino acid transporters and biosynthesis, translation, metal transporters, and fatty acid biosynthesis.

In order to gain insight into the functions of the LGG differentially expressed genes regulated by YAMC-CM, we analyzed these genes using the Clusters of Orthologous Groups (COGs) database. The COG database is a comprehensive tool for genome-scale characterization of bacterial gene products that is based on more than 1,100 bacteria genomes and classifies genes into 25 functional categories (Voss et al., 2015; Galperin et al., 2021). COG analysis of the 231 YAMC-CM upregulated genes identified the top 10 COG categories to be amino acid transport and metabolism (E, 42 genes), translation including ribosome structure and biogenesis (J, 32 genes), inorganic ion transport and metabolism (P, 23 genes), lipid transport and metabolism (I, 15 genes), cell wall/membrane/envelope biogenesis (M, 12 genes), replication, recombination and repair (L, 8 genes), carbohydrate transport and metabolism (G, 7 genes), transcription (K, 6 genes), defense mechanisms (V, 5 genes), and no functional prediction (S, 55 genes; Figure 2A).

We next determined the most biologically relevant genes of the statistically significant upregulated genes of LGG by YAMC-CM. We calculated the difference in expression between the YAMC-CM and RPMI control group averages for each of the 231 significantly upregulated genes (FDR < 0.05) to identify the top 100 differentially expressed genes. Categorization of the top 100 differentially expressed LGG genes (based on the difference in absolute abundance) by Gene Ontology (GO) biological process and molecular function was then performed. The top seven categories (more than five genes in each category) identified by this analysis include amino acid transporters and biosynthesis, translation, metal transporters, fatty acid biosynthesis, common ATP-binding cassette (ABC) transporter components, cell division-DNA replication, cell wall biosynthesis (Lipoteichoic acid) and modification (Figure 2B). The actual expression values (average normalized CPM) for the top genes in the categories of amino acid transporters and biosynthesis, translation, metal transporters, and fatty acid biosynthesis are displayed in the radar plots (Figure 2C), demonstrating greater absolute abundance in the YAMC-CM group than the RPMI group. The top 100 upregulated genes with significant alteration in LGG cultured with YAMC-CM (Figure 2B) are shown in Supplementary Table 1.

These data suggest that YAMC can upregulate several bioactivities of LGG. YAMC-CM upregulated 44 genes encoding membrane transporters for amino acids, metals, and sugars. Among these genes, there are 27 genes encoding ABC transporters (Supplementary Table 1). This result indicates that the major activity associated with YAMC-CM-regulated genes is nutrient acquisition. YAMC-CM-regulated genes are also associated with membrane biosynthesis in LGG by providing fatty acid resources and a cell wall component, lipoteichoic acid. Within the fatty acid biosynthesis category, the 10 upregulated genes are all linked to fatty acid synthesis, including functions such as fatty acid elongation, malonate to Acyl carrier protein (ACP) transfer and acetyl-CoA carboxyl carrier protein. Further, YAMC-CM upregulated genes for lipoteichoic acid biosynthesis, such as several D-alanyl-lipoteichoic acid biosynthesis proteins (Supplementary Table 1). A large group of genes involved in translation are up-regulated by YAMC-CM. Interestingly, the increase in several amino acid tRNA ligases in RNA processing further suggests that YAMC-CM enhances amino acid biosynthesis at the translational level (Supplementary Table 1).

**Figure 3:**
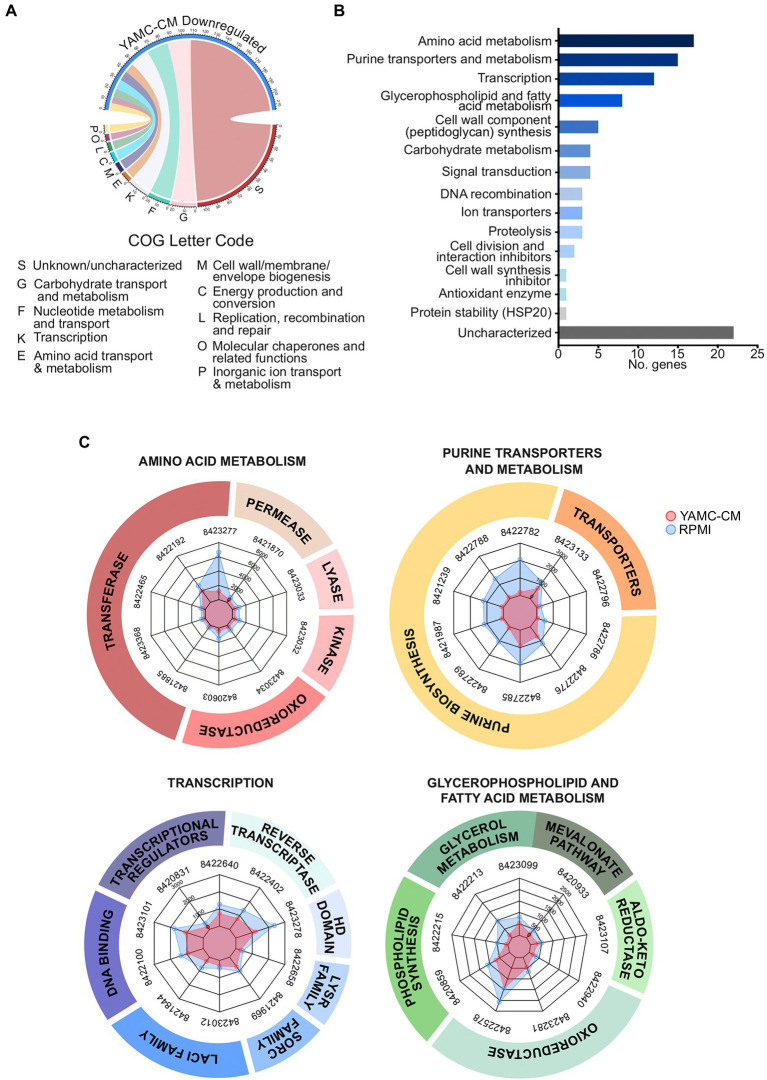
Identification of 235 differentially downregulated LGG genes by YAMC conditioned medium (YAMC-CM). **(A)** The 235 downregulated genes (FDR < 0.05) were classified by the COG system and then **(B)** sorted by abundance differential with the RPMI group to identify the top 100 YAMC-CM downregulated genes by expression difference, which were then categorized by GO biological process and molecular function. **(C)** Radar plots comparing the YAMC-CM and RPMI mean group expression (in CPM) for the top 9–10 most abundant genes for the enriched categories amino acid metabolism, purine transporters and metabolism, transcription, and glycerophospholipid and fatty acid metabolism.

YAMC-CM treatment resulted in significantly repressing the expression of 235 genes in LGG (FDR < 0.05). The top 10 COG categories that these downregulated genes are associated with carbohydrate metabolism and transport (G, 22 genes), nucleotide metabolism and transport (F, 20 genes), transcription (K, 18 genes), amino acid metabolism and transport (E, 9 genes), cell wall structure and biogenesis and outer membrane (M, 9 genes), energy production and conversion (C, 9 genes), replication, recombination and repair (L, 8 genes), molecular chaperones and related functions (O, 7), inorganic ion transport and metabolism (P, 6 genes), and no functional prediction (S, 107 genes; Figure 3A).

Using the same analysis process for upregulated genes, the top 100 most extreme down regulated genes (by actual expression values) were further analyzed by GO biological process and molecular function (Figure 3B). Genes in the top five categories (more than 5 genes in each category) are amino acid metabolism, purine transporters and metabolism, transcription, glycerophospholipid and fatty acid metabolism, and cell wall component (peptidoglycan) synthesis. The abundance of the top 10 highest downregulated genes in these five categories was plotted in radar plots (normalized CPM) to illustrate the conserved difference in expression between the YAMC-CM groups as compared to the control RPMI group (Figure 3C). All of the 100 top downregulated genes with significant alteration in LGG cultured with YAMC-CM (Figure 3B) are presented in Supplementary Table 2.

The major down-regulated genes in LGG by YAMC-CM are linked to catabolism of amino acids, purines, glycerophospholipid and fatty acids, and carbohydrates (Supplementary Table 2). This evidence may be related to the metabolic regulation mechanism in response to the availability of nutrients in bacteria (Heinemann and Sauer, 2010; Kotte et al., 2010; Chubukov et al., 2014). This data also suggests that the downregulation of genes involved in metabolism is not the direct effect of IEC-derived components on LGG, but more of a secondary response to the sufficient nutrients by LGG.

### IEC-derived components promote LGG growth

**Figure 4:**
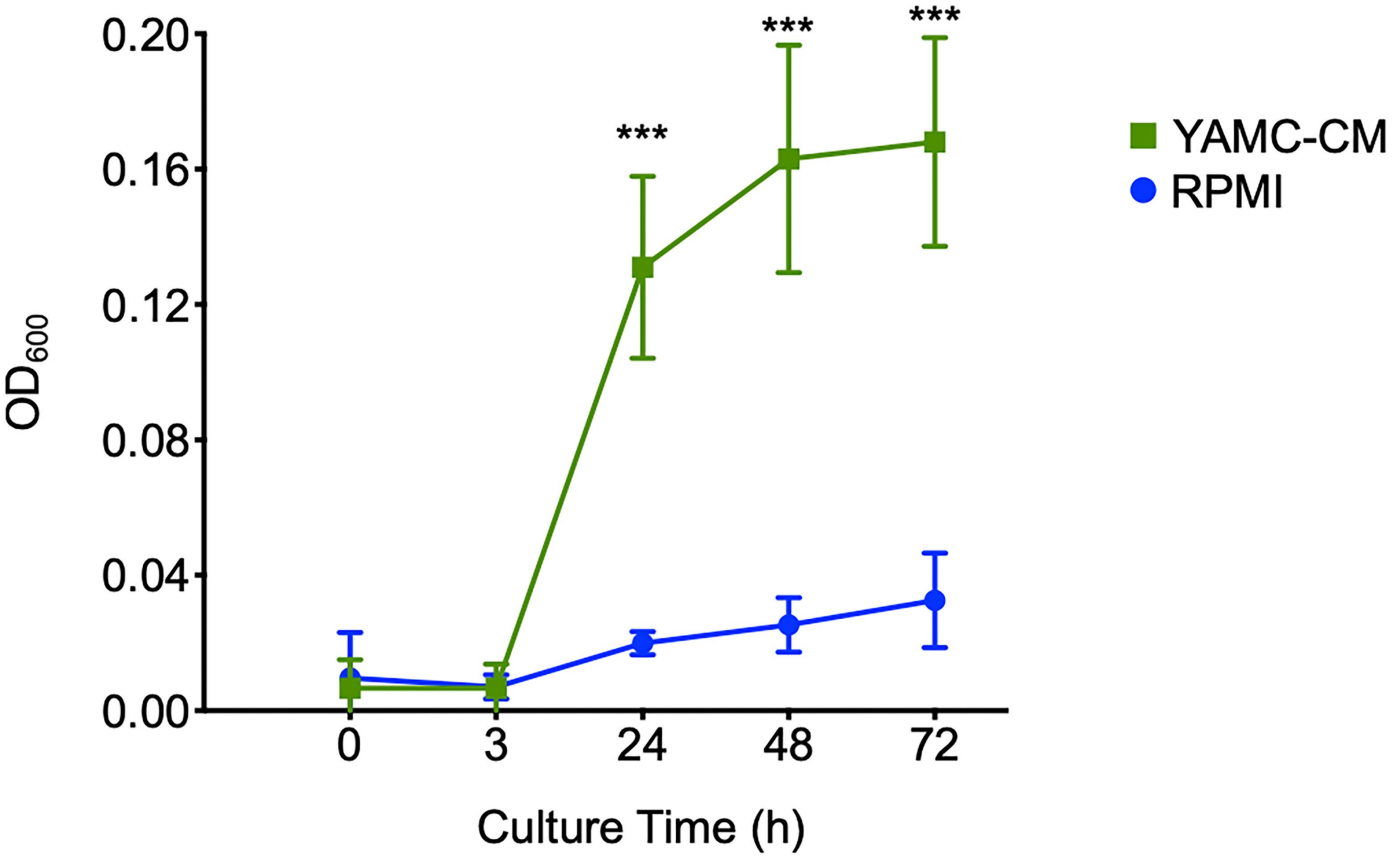
Intestinal epithelial cell-derived components (IEC-DCs) increase LGG growth *in vitro*. LGG was cultured in YAMC-CM (24-h cell culture) or RPMI. The LGG growth curve is shown. ***p < 0.01 compared to RPMI at the same culture time.

As the molecular mechanisms upregulated by YAMC-CM suggested an influence on LGG growth, we tested the functional effect of YAMC-CM on LGG growth *in vitro*. Compared to LGG growth in RPMI medium, YAMC-CM had a significant effect on promoting LGG growth in the exponential phase (at 24 and 48 h of culture), and maintained LGG number significant higher in stationary phase (at 72 h of culture; Figure 4). Taken together, predicted functions based on YAMC-CM regulation of the LGG transcriptome are consistent with the phenotypic effect of YAMC-CM on LGG growth.

## Discussion

Although the mechanisms mediating the beneficial effects of the gut microbiota on the host have been widely studied, mechanistic understanding of the influence of the host factors on microbial growth, adaptation and function is insufficient. It is well-known that commensal bacteria exert beneficial effects on IECs to maintain the mucosal barrier and intestinal homeostasis (Thomas and Versalovic, 2010; Yan and Polk, 2020). Interesting findings from our previous studies demonstrated that IEC-derived components upregulated the function of LGG for protecting IECs against proinflammatory insults and ameliorating colitis (Yang et al., 2019). Here, we have defined previously unrecognized molecular effects of IEC-derived components on LGG. By performing functional analysis of transcriptome data, we identified expression levels of numerous genes in LGG in response to IEC-derived components. Predominant gene profiles upregulated by YAMC-CM are associated with the increase in nutrient acquisition by enriching amino acid, metal, and sugar transporters and amino acid and fatty acid biosynthesis. In addition, genes upregulated by YAMC-CM are involved in cell wall biosynthesis and cell division. One possible outcome of these activities is to promote bacterial growth. Our data further confirmed that YAMC-CM promoted LGG growth. Adherence to the mucus layer on the surface of intestinal epithelial monolayer is a physiological characteristic of bacteria that is necessary for colonization and survival in the gastrointestinal tract. Our analysis of the YAMC-CM-regulated LGG transcriptome did not predict any impact of YAMC-CM on LGG adhesion properties. This was confirmed by the finding that LGG cultured in YAMC-CM and in RPMI medium showed similar adhesion ability to the mucin surface (*p* > 0.05; Supplementary Figure 1). These results provide mechanistic insight on the regulation of the gut microbiota community by IECs.

We previously performed iTRAQ based proteomic analysis of LGG protein levels regulated by YAMC-CM (Yang et al., 2019). Several up-regulated proteins in LGG by YAMC-CM are encoded by genes identified in this study, including transporters, enzymes for amino acid synthesis, and cell wall synthesis. This evidence indicates that the effects of IECs on the gut microbiota may be through transcriptional and translational regulation.

It should be noted that the bacterial transcriptome is altered according to the bacteria growth status. It has been reported that gene expression in the exponential phase was different from that in the stationary phase; for example, genes associated with glucose fermentation in the growth phase were shifted to galactose utilization in the stationary phase (Laakso et al., 2011). In this study, based on the cell culture curve (Figure 4), RNA was isolated from LGG at the transition from the exponential to the stationary phase. Therefore, we expect we could reveal the transcriptomic profile of LGG regulated by YAMC-CM in both exponential and stationary phases. In addition, the integration of metabolism and environmental conditions by bacteria leads to the alteration of gene expression and the modulation of protein activity. Thus, changes in bacterial gene expression are associated with the metabolic status in different environment conditions (Chubukov et al., 2014). Our results suggest the regulatory effect of IEC-derived components on LGG gene expression under basal growth condition (in RPMI 1640 medium). The regulatory effects of IEC-derived components on LGG in different nutrient statuses is worth further investigation.

In addition to providing tensile strength and maintaining cell shape, the cell wall surrounding gram positive bacteria plays significant roles in cell grow, nutrient uptake, and protection against insults. In our study YAMC-CM regulated genes involved in cell wall biosynthesis in LGG in different manners. Lipoteichoic acid (LTA) is an important cell wall polymer found in gram-positive bacteria; YAMC-CM regulated genes involved in biosynthesis of LTA (Supplementary Table 1). Interestingly an inhibitor of cell wall synthesis, glycopeptide antibiotics resistance protein, was repressed (Supplementary Table 2). On the other hand, YAMC-CM downregulated genes are associated with the synthesis of peptidoglycan (Supplementary Table 2). Peptidoglycan interacts with other macromolecules on the outside of cell membranes and contributes to preserving cell integrity. Elongation and septation of peptidoglycan are complicated and requires coordination with other cell syntheses and the cell cycle status (van Heijenoort, 2001; Chapot-Chartier and Kulakauskas, 2014); thus it is difficult to predict the effects of alteration of genes on peptidoglycan biosynthesis *in vivo*.

Results from this study suggest that not all alterations of the transcriptome in LGG are directly regulated by IEC-derived components. Bacteria are exposed to a constantly changing physical and chemical environment. Thus, bacteria develop sophisticated mechanisms for the regulation of both catabolic and anabolic pathways to adapt to the ecological situation, such as the availability of nutrients (Heinemann and Sauer, 2010; Kotte et al., 2010; Chubukov et al., 2014). In the presence of sufficient nutrients, the production of metabolic enzymes may be inhibited in bacteria. Notably, transcriptional control represents one of the steps for the regulation of enzymes in metabolism in bacteria (Kochanowski et al., 2013). Thus, the YAMC-CM-repressed genes which encode enzymes for metabolism in LGG might represent secondary effects under the impact of YAMC-CM-induced nutrient acquisition, but not from the direct effects of YAMC-CM on LGG.

In addition to the regulation of LGG at the transcriptome level, IECs may exert their impact on LGG *via* other programs in LGG. For example, we previously found that IEC-derived components stimulated production of a functional protein, p40, by LGG (Yang et al., 2019). Consistent with our previous finding that YAMC-CM did not affect p40 gene expression in LGG by qPCR analysis (Yang et al., 2019), p40 gene expression was not affected by IEC-derived components by RNA sequencing. Other potential effects of IEC-derived components may come from regulation of protein stability and secretion. For example, IEC-derived components upregulated one molecular chaperone, a heat shock protein (HSP), GroEL (Supplementary Table 1). HSPs promote protein folding, enabling the stability and transportation of client proteins, resulting in growth, metabolism, transport functions, and protein synthesis and stability in Lactobacillus (Rossi et al., 2016). Therefore, the impact of IECs on the gut microbiota may be at multiple functional levels.

Our previous works (Yang et al., 2019) have demonstrated that intestinal epithelial cell-secreted extracellular vesicles (IEC-EVs) are up-taken by LGG. These results suggest that IEC-EVs may functionally mediate the communication between IECs and LGG. Among the protein cargos in IEC-EVs identified in our study, a molecular chaperone, heat shock protein (HSP) 90, particularly HSP90β, is a potential target. HSP90 is highly conserved from bacteria to mammals and displays functional overlap in protein folding, enabling the stability and transportation of client proteins (Chen et al., 2006; Johnson, 2012). Regulating gene transcription represents one of mechanisms of HSP90 client proteins. Client proteins of HSPs in Lactobacilli regulate growth, metabolism, transport functions, and protein synthesis (Rossi et al., 2016). Therefore, potential functional components could be protein cargos within IEC-EVs. Although we do not exclude other cargos in IEC-IEVs, our future studies will be focused on investigating the contribution of HSP90β in IEC-EVs to the molecular regulatory effects on the gut microbiota by IECs.

In summary, our functional analysis identified novel transcriptional targets of IEC-derived components in LGG that are associated with nutrient acquisition and enable LGG growth. These findings demonstrate how LGG adapts on a molecular transcriptional level to environmental signals from the host, depicting the intimate transcriptional regulatory control the host exerts over the growth of LGG, which is made lucid through YAMC-CM initiated upregulation of key LGG genes involved in amino acid transport and biosynthesis, translation, metal transport, and fatty acid synthesis, all of which are necessary for LGG growth. Indeed, this previously unrecognized transcriptional impact of IECs on commensal bacteria has important applications for elucidating the mechanisms mediating the mutual interactions between the gut microbiota and the host.

## Materials and methods

### Preparation of young adult mouse colonic epithelial cell-conditioned medium and LGG treatment

YAMC cell lines were generated from the colon from C57BL/6J mice harboring a thermolabile mutation (tsA58) under the control of an IFN-γ-inducible H-2Kb promoter and a temperature-sensitive simian virus 40 large T antigen (immortomouse) (Whitehead et al., 1993). YAMC cells were cultured in RPMI 1640 medium supplemented with 10% FBS, 5 U/ml of mouse IFN-γ, 100 U/ml penicillin and streptomycin, 5 μg/ml insulin, 5 μg/ml transferrin, and 5 ng/ml selenous acid at 33°C (permissive temperature) under 5% CO_2_. For preparation of YAMC-CM, YAMC cells with confluency at 80–90% were cultured in RPMI 1640 medium only (without FBS, IFN-γ, penicillin and streptomycin, insulin, transferrin, or selenous acid) for 24 h at 37°C (non-permissive temperature). Then culture supernatants were processed through successive differential centrifugation at 300 × *g* for 10 min, 2,000 × *g* for 20 min, and 10,000 × *g* for 30 min. The supernatants were saved as YAMC-CM. Cell numbers were counted to normalize YAMC-CM at 2 × 10^5^ cells/ml. To exclude the influence of apoptotic and necrotic fragments on CM, cells were maintained in culture with a survival rate higher than 90%.

LGG (American Type Culture Collection 53,103) were prepared from cultures in *Lactobacillus* MRS broth at 37°C with OD_600_ = 0.5 to 1.0. LGG was cultured with YAMC-CM (2 × 10^7^ CFU/ml) at 37°C for 24 h. Culture of LGG in RPMI was used as a control. RPMI 1640 medium contains amino acids, vitamins, glucose (2 g/L), glutathione, HEPES, and inorganic salts, which provide basal levels of nutrients for LGG growth. There is no FBS in RPMI 1640 medium.

### RNA extraction, library preparation and sequencing

Total RNA was extracted from LGG cells cultured with YAMC-CM or RPMI. Each group contained three LGG samples from independent culture experiments. LGG was homogenized in 600 μl QIAzol (Qiagen) and 500 μl of 2.0 mm zirconium oxide beads (Next Advance, Inc. Cat: ZROB05) using a Bullet Blender homogenizer (BB24-AU, Next Advance, Inc). While homogenizing the samples, temperature was maintained at 4°C by using the dry ice cooling system in the Bullet Blender. The homogenate was treated with 100 μl of genomic DNA Eliminator solution (Qiagen) to remove the genomic DNA. Next, 180 μl of chloroform was added to the samples for phase separation. The total RNA in the aqueous phase was then purified using RNeasy Mini spin columns as recommend by the Qiagen RNeasy protocol. RNA integrity and RNA quantification were assessed using an Agilent Bioanalyzer RNA 6000 Nano/Pico Chip (Agilent Technologies, Palo Alto, CA, United States). Eukaryotic ribosomal RNAs (rRNA) were depleted using the NEBNext rRNA Depletion Kit (Human/Mouse/Rat, Cat: E6310X). After rRNA depletion, the samples were checked by Agilent Bioanalyzer RNA 6000 Nano/Pico Chip to ensure depletion of the 18S and 28S ribosomal peaks. Next, Illumina sequencing libraries were made using the NEBNext Ultra II RNA Library Prep Kit (NEB #E7775). The quality of the libraries was assessed using an Agilent Bioanalyzer DNA High Sensitivity chip. The libraries were then sequenced on an Illumina NovaSeq6000 platform (S4 flow cells run) with 2 × 150 base pair reads, with a sequencing depth of ~20 million paired-end reads per sample.

### Quantification and statistical analysis

For preprocessing and quality control of NGS data, adapter removal and quality-based trimming of the raw reads were performed using Trimmomatic v0.39 (Bolger et al., 2014). Trimmed reads shorter than 50 nt were discarded. Low complexity reads were discarded using *bbduk* from bbtools (Bushnell et al., 2017) with entropy set at 0.7 (BBMap – Bushnell B.).^1^ LGG reads were mapped to *Lacticaseibacillus rhamnosus* GG reference genome NC_013198 using HISAT2 (Kim et al., 2015). The read counts for each gene feature was quantified using HTSeq (Anders et al., 2015). The feature counts of all the samples were combined into a single matrix using a custom R script. Differential expression analysis was performed by comparing YAMC-CM and RPMI groups using the DESeq2 package (Love et al., 2014). Genes with an adjusted value of *p* < 0.05 were treated as differentially expressed.

For functional analysis of LGG genes, Clusters of Orthologous Groups (COGs) Database classification was used for phylogenetic functional classification of LGG microbial proteins (Voss et al., 2015; Galperin et al., 2021). Briefly, protein FASTA files were obtained from NCBI and the corresponding COG letter was identified using EggNog 5.0.0 (Huerta-Cepas et al., 2019).^2^ Protein function was further determined based on GO biological process and GO molecular function classifications.

### LGG growth

To identify the effects of YAMC-CM on LGG growth *in vitro*, LGG was cultured in YAMC-CM or RPMI (control) for total 72 h. OD_600_ was recorded at 0, 3, 24, 48, and 72 h to generate a growth curve. This experiment was repeated 3–5 times.

### Statistical analysis

LGG growth curve data is presented as mean ± standard deviation (SD). Statistical significance was determined using multiple unpaired *t*-tests for comparing data from two samples using GraphPad Prism 8.0 (GraphPad Software, Inc., San Diego, CA, United States). *p < 0.10, **p < 0.05, ***p < 0.01.

## Data availability statement

The data presented in the study are deposited in the NCBI SRA database repository, accession number PRJNA 907027 (https://www.ncbi.nlm.nih.gov/sra/PRJNA907027).

## Author contributions

KS and SR performed RNA sequencing, transcriptome data acquisition and the data analysis. KS, SR, SD, RO, and FY conceived and designed the study, performed data analysis, and prepared the manuscript. All authors contributed to the article and approved the submitted version.

## Funding

This work was supported by the National Institutes of Health (NIH) grants, R01DK081134-12 (FY) and R21AI149262 (SD), the Crohn’s & Colitis Foundation Senior Research Award (FY), Training Grant in Gastroenterology (T32DK007673-21) (KS), and Vanderbilt University Medical Center’s Digestive Disease Research Center supported by NIH grant P30DK058404.

## Conflict of interest

The authors declare that the research was conducted in the absence of any commercial or financial relationships that could be construed as a potential conflict of interest.

## Publisher’s note

All claims expressed in this article are solely those of the authors and do not necessarily represent those of their affiliated organizations, or those of the publisher, the editors and the reviewers. Any product that may be evaluated in this article, or claim that may be made by its manufacturer, is not guaranteed or endorsed by the publisher.

## Footnotes

^1^ sourceforge.net/projects/bbmap/

^2^ http://eggnog5.0.0
