# Supplementary material for: Intestinal epithelial cell-derived components regulate transcriptome of Lactobacillus rhamnosus GG
Source: Front Microbiol. 2023 Jan 4;13:1051310. doi: 10.3389/fmicb.2022.1051310 (PMC9846326; doi:10.3389/fmicb.2022.1051310)
Supplement: Supplementary file 1 [file Data_Sheet_1.PDF]

Supplementary Table 1. LGG transcriptome up-regulated by YAMC-CM.

Gene name	Gene ID	Locus tag	Log_2FC	FDR
Amino acid transporters and biosynthesis				
Proline/glycine betaine ABC transporter permease	8422175	LGG_RS10325	2.048982416	4.63E-23
Glycine/betaine ABC transporter	8422692	LGG_RS10320	1.968101867	1.24E-21
Glycine/betaine/L-proline ABC transporter ATP-binding protein	8422174	LGG_RS10330	1.970898017	1.68E-21
Amino acid permease	8422222	LGG_RS02865	1.547131074	4.86E-14
Aspartate kinase	8422432	LGG_RS00560	1.073801453	6.47E-07
Peptide ABC transporter substrate-binding protein	8422588	LGG_RS09365	0.958006919	5.07E-06
Diaminopimelate epimerase	8423031	LGG_RS00565	0.97396263	8.35E-06
Peptide ABC transporter substrate-binding protein	8422654	LGG_RS01720	0.942345893	1.62E-05
Branched-chain amino acid ABC transporter permease	8422417	LGG_RS01505	0.834365037	0.000307466
Pyroglutamyl-peptidase I	8420570	LGG_RS01160	0.74030122	0.000738589
Aspartate-semialdehyde dehydrogenase	8422029	LGG_RS00570	0.732145171	0.001367551
Peptide ABC transporter substrate-binding protein	8422589	LGG_RS09950	0.674506485	0.001765862
ABC transporter permease subunit	8422201	LGG_RS13685	0.629974141	0.005459274
Amino acid ABC transporter ATP-binding protein	8421758	LGG_RS13675	0.633588977	0.005856519
C1 family peptidase	8421511	LGG_RS11230	0.623967809	0.007619038
Asparagine synthase	8421426	LGG_RS10450	0.587607788	0.009078619
C69 family dipeptidase	8421004	LGG_RS05560	0.597203714	0.010916512
ABC transporter permease subunit	8422207	LGG_RS13680	0.577630494	0.011741486
Aminopeptidase P family protein	8422082	LGG_RS08110	0.591358894	0.012625842
Oligopeptide ABC transporter substrate-binding protein	8422863	LGG_RS07940	0.51416336	0.024520872
C1 family peptidase	8422635	LGG_RS11235	0.499045854	0.032996861
Serine/threonine transporter SstT	8422750	LGG_RS02860	0.502777326	0.040403637
Metal transporters				
Divalent metal cation transporter	8421560	LGG_RS11535	2.280604366	5.70E-29
Metal ABC transporter ATP-binding protein	8422505	LGG_RS11585	1.678543993	1.33E-15
Metal ABC transporter permease	8422506	LGG_RS11580	1.523274703	4.62E-14
Manganese ABC transporter substrate-binding protein	8422018	LGG_RS11575	1.394331662	4.37E-12
ABC transporter permease	8422595	LGG_RS09940	1.024749663	5.77E-07
ABC transporter permease	8422591	LGG_RS09360	1.047774897	1.29E-06
Cadmium-translocating P-type ATPase	8421727	LGG_RS13415	0.99738299	1.62E-06
ABC transporter permease	8421310	LGG_RS09355	0.940484323	1.63E-05
ABC transporter permease	8422592	LGG_RS09945	0.875994401	3.13E-05
Cation:proton antiporter	8423359	LGG_RS03480	0.597173578	0.011870646
ABC transporter permease	8422590	LGG_RS07950	0.571806148	0.012287001

Sugar transporters				
PTS sugar transporter subunit IIC	8421108	LGG_RS06960	0.625831782	0.005990872
PTS fructose transporter subunit IIABC	8421659	LGG_RS12645	0.604932914	0.007946914
Common ABC transporter components				
ABC transporter ATP-binding protein	8421335	LGG_RS09570	1.774999824	7.95E-15
ABC transporter permease	8421873	LGG_RS09565	1.563283219	2.58E-14
ABC-F family ATP-binding cassette domain-containing protein	8420590	LGG_RS01455	1.557342049	3.29E-13
ABC transporter ATP-binding protein	8421309	LGG_RS09340	1.180517806	2.54E-08
ABC transporter ATP-binding protein	8422597	LGG_RS09345	0.971991292	4.64E-06
ATP-binding cassette domain-containing protein	8422247	LGG_RS09930	0.877828782	2.68E-05
ABC transporter ATP-binding protein	8422598	LGG_RS09935	0.855184769	4.69E-05
ABC transporter permease	8422143	LGG_RS04310	0.733916988	0.001209472
ATP-binding cassette domain-containing protein	8420754	LGG_RS03010	0.704431081	0.001299632
ABC transporter permease	8423322	LGG_RS04610	0.59178036	0.013235145
Translation-RNA processing and ribosome biosynthesis				
50S ribosomal protein L10	8422884	LGG_RS10910	1.413790073	3.84E-12
Tyrosine--tRNA ligase	8421351	LGG_RS09715	1.284516054	9.36E-09
50S ribosomal protein L7/L12	8422716	LGG_RS10905	1.16948945	2.70E-08
Leucine--tRNA ligase	8420845	LGG_RS04085	1.000198857	1.66E-06
Phenylalanine--tRNA ligase subunit beta	8421207	LGG_RS08255	0.948045613	9.35E-06
Aspartate--tRNA ligase	8422500	LGG_RS07495	0.857539336	8.05E-05
Isoleucine--tRNA ligase	8422048	LGG_RS06200	0.75369875	0.000497072
50S ribosomal protein L13	8422922	LGG_RS11705	0.758096573	0.000590767
Alanine--tRNA ligase	8420806	LGG_RS03615	0.771843992	0.000837561
Ribosome biogenesis GTPase Der	8422914	LGG_RS06655	0.66097807	0.004721375
Valine--tRNA ligase	8421050	LGG_RS06060	0.635283179	0.005459274
30S ribosomal protein S9	8421582	LGG_RS11700	0.595856803	0.00953064
Ribonuclease J	8421065	LGG_RS06290	0.585696504	0.009967663
Threonine--tRNA ligase	8421219	LGG_RS08435	0.606698491	0.010690509
Elongation factor G	8421589	LGG_RS11910	0.554585275	0.01341478
50S ribosomal protein L11	8422875	LGG_RS10970	0.55871306	0.01754092
50S ribosomal protein L1	8421481	LGG_RS10965	0.52927336	0.021322661
Methionine--tRNA ligase	8421629	LGG_RS12360	0.520911806	0.024921085
tRNA (uridine/cytosine/5-carboxymethylaminomethyluridine-2’-O)-methyltransferase TrmL	8420855	LGG_RS04220	0.538712192	0.02599506
30S ribosomal protein S16	8423262	LGG_RS07900	0.488013028	0.042401142
Fatty acid biosynthesis				

Acetyl-CoA carboxylase biotin carboxylase subunit	8421829	LGG_RS10165	1.457717162	1.05E-13
Acetyl-CoA carboxylase carboxyl transferase subunit alpha	8421950	LGG_RS10155	1.485201167	1.05E-13
Ketoacyl-ACP synthase III	8421834	LGG_RS10205	1.05567738	1.85E-07
Beta-ketoacyl-ACP synthase II	8421393	LGG_RS10180	1.557196	2.26E-15
Acetyl-CoA carboxylase biotin carboxyl carrier protein	8422104	LGG_RS10175	1.628472649	2.93E-15
3-hydroxyacyl-ACP dehydratase FabZ	8421828	LGG_RS10170	1.392860965	3.46E-11
3-oxoacyl-ACP reductase FabG	8422099	LGG_RS10185	1.627818233	3.46E-16
ACP S-malonyltransferase	8422101	LGG_RS10190	1.594939513	6.25E-16
Hypothetical protein	8421827	LGG_RS10160	1.48346391	7.01E-14
Beta-hydroxyacyl-ACP dehydratase	8422454	LGG_RS10215	0.944651509	7.52E-06
Cell division-DNA replication				
DNA topoisomerase IV subunit A	8422617	LGG_RS06805	0.830729714	0.000131371
ATP-dependent DNA helicase	8423245	LGG_RS09515	0.757200596	0.000476136
DEAD/DEAH box helicase	8421835	LGG_RS12145	0.702047673	0.001100931
ATP-dependent DNA helicase RecG	8422683	LGG_RS07975	0.727527101	0.001164332
Cell division ATP-binding protein FtsE	8422703	LGG_RS04305	0.727202657	0.001704024
DNA topoisomerase IV subunit B	8423271	LGG_RS06800	0.604221632	0.009833954
ATP-dependent DNA helicase	8421645	LGG_RS12505	0.580175336	0.014659642
Cell wall biosynthesis (Lipoteichoic acid) and modification				
D-alanine--poly(phosphoribitol) ligase subunit DltA	8422055	LGG_RS03665	1.193077204	3.95E-09
D-alanyl-lipoteichoic acid biosynthesis protein DltB	8422050	LGG_RS03670	1.053092321	3.49E-07
D-alanyl-lipoteichoic acid biosynthesis protein DltD	8422052	LGG_RS03680	0.995826782	1.39E-06
C40 family peptidase	8421350	LGG_RS09710	0.975256923	7.52E-06
Bifunctional lysylphosphatidylglycerol flippase/synthetase MprF	8422949	LGG_RS10880	0.75920913	0.000660859
Glycosyl transferase	8420900	LGG_RS04790	0.517032403	0.034647344
ATP synthesis				
F0F1 ATP synthase subunit A	8421011	LGG_RS05650	0.597693354	0.009057447
Protein stability				
Chaperonin GroEL	8421461	LGG_RS10730	0.811625766	0.000144208
Stress response				
MarR family transcriptional regulator	8422102	LGG_RS10210	0.970527072	6.13E-06
Universal stress protein	8421413	LGG_RS10340	0.495623592	0.038650363
Nitrogen metabolism				
2-nitropropane dioxygenase	8421394	LGG_RS10195	1.074113727	1.03E-07

Uncharacterized				
Hypothetical protein	8422609	LGG_RS11545	1.206108412	1.33E-07
DUF979 family protein	8420566	LGG_RS01145	1.035540114	1.66E-06
DUF969 domain-containing protein	8420565	LGG_RS01140	0.974849475	5.90E-06
Hypothetical protein	8421924	LGG_RS01420	0.815080448	0.000155247
Endolytic transglycosylase MltG	8423088	LGG_RS08250	0.778528315	0.000740122
DUF805 domain-containing protein	8420535	LGG_RS00930	0.701434143	0.001790111
Flotillin family protein	8421868	LGG_RS10370	0.489943293	0.038996701

Supplementary Table 2. LGG transcriptome down-regulated by YAMC-CM.

Gene name	Gene ID	Locus tag	Log_2FC	*FDR*
**Amino acid metabolism**				
PLP-dependent aminotransferase family protein	8423277	LGG_RS12610	-2.473902353	4.33E-37
FAD-dependent oxidoreductase	8420603	LGG_RS01555	-1.665889898	5.05E-17
Aspartate kinase	8423032	LGG_RS10365	-1.294487711	1.67E-10
Threonine synthase	8423033	LGG_RS10355	-1.180145778	8.52E-09
SDR family NAD(P)-dependent oxidoreductase	8422501	LGG_RS05845	-1.340734084	1.86E-08
Homoserine kinase	8423368	LGG_RS10350	-1.181708878	2.31E-08
Homoserine dehydrogenase	8423034	LGG_RS10360	-1.146157836	2.84E-08
Aspartate ammonia-lyase	8423151	LGG_RS13730	-1.059577883	3.52E-06
N-acetyltransferase	8422308	LGG_RS04825	-0.910604079	0.000109791
Homoserine O-succinyltransferase	8421885	LGG_RS02685	-0.845357942	0.00015651
Cysteine synthase A	8422465	LGG_RS02690	-0.784745007	0.000366327
FAD/NAD(P)-binding protein	8422940	LGG_RS01040	-0.790074069	0.000542134
Amino acid permease	8421870	LGG_RS02910	-0.692408114	0.001841707
Pyruvate oxidase	8421250	LGG_RS08835	-0.663248297	0.005878631
Alanine racemase	8421609	LGG_RS12135	-0.624305834	0.009986579
CBS domain-containing protein	8422214	LGG_RS03705	-0.564142444	0.021092119
Glutamine--fructose-6-phosphate transaminase	8422192	LGG_RS04715	-0.528103545	0.021267213
Purine transporters and metabolism				
Amidophosphoribosyltransferase	8422788	LGG_RS08705	-3.66989176	1.89E-66
Phosphoribosylformylglycinamidine synthase subunit PurL	8422782	LGG_RS08710	-3.473646702	4.86E-63
Phosphoribosylformylglycinamidine cyclo-ligase	8422789	LGG_RS08700	-3.606843892	6.22E-63
Bifunctional phosphoribosylaminoimidazolecarboxamide formyltransferase/IMP cyclohydrolase	8421239	LGG_RS08690	-3.440378223	2.05E-59
Phosphoribosylamine--glycine ligase	8421987	LGG_RS08685	-3.392224068	5.20E-58
Phosphoribosylglycinamide formyltransferase	8422783	LGG_RS08695	-3.721923046	1.16E-49
Phosphoribosylformylglycinamidine synthase subunit PurQ	8422786	LGG_RS08715	-3.152962705	2.04E-45
Phosphoribosylaminoimidazolesuccinocarboxamide synthase	8422791	LGG_RS08725	-3.453719812	2.59E-40
5-carboxyamino)imidazole ribonucleotide synthase	8422779	LGG_RS08730	-2.72269768	5.70E-29
Uracil transporter	8422796	LGG_RS07000	-1.586995938	2.20E-12
Formate--tetrahydrofolate ligase	8422429	LGG_RS07020	-1.064596526	1.78E-07
Adenylosuccinate lyase	8422785	LGG_RS05160	-1.039464664	3.49E-07
ATP-grasp domain-containing protein	8422776	LGG_RS05155	-0.787847022	0.000241516
Purine permease	8423133	LGG_RS05150	-0.618122631	0.006064147
IMP dehydrogenase	8420573	LGG_RS01220	-0.52309527	0.023392107
Transcriptional regulators				
LacI family transcriptional regulator	8421844	LGG_RS10135	-1.30801323	1.71E-10
Sigma-70 family RNA polymerase sigma factor	8420831	LGG_RS03975	-1.093123365	4.24E-07

HD domain-containing protein	8423278	LGG_RS08270	-0.945663328	4.46E-06
Reverse transcriptase	8422402	LGG_RS09415	-0.775263355	0.000303872
Zinc ribbon domain-containing protein	8421402	LGG_RS10265	-0.8198475	0.000378874
LytR family transcriptional regulator	8422640	LGG_RS01410	-0.751244042	0.000496909
Helix-turn-helix domain-containing protein	8423101	LGG_RS10630	-0.667735684	0.002326372
Helix-turn-helix domain-containing protein	8422100	LGG_RS04250	-0.529231344	0.022627679
LysR family transcriptional regulator (virulence, metabolism, quorum sensing and motility)	8422658	LGG_RS06820	-0.52295456	0.025615096
Spx/MgsR family RNA polymerase-binding regulatory protein	8422341	LGG_RS09790	-0.525816183	0.035185722
LacI family transcriptional regulator	8423012	LGG_RS02020	-0.508677344	0.037496785
SorC family transcriptional regulator	8421969	LGG_RS04460	-0.511190314	0.038810834
Glycerophospholipid and fatty acid metabolism				
Glycerol kinase GlpK	8422213	LGG_RS03035	-2.437006203	6.56E-33
Type 1 glycerol-3-phosphate oxidase	8422215	LGG_RS03030	-2.440921378	5.78E-30
Phosphomevalonate kinase	8420933	LGG_RS05075	-0.908536434	3.76E-05
CDP-diacylglycerol--glycerol-3-phosphate 3-phosphatidyltransferase	8420859	LGG_RS04255	-0.697223049	0.001492611
Glyceraldehyde 3-phosphate reductase	8423107	LGG_RS12565	-0.604866518	0.010029187
Glycerophosphoryl diester phosphodiesterase	8423099	LGG_RS08625	-0.551584219	0.018182405
D-2-hydroxyacid dehydrogenase	8422578	LGG_RS00785	-0.519928526	0.024442405
NAD-dependent succinate-semialdehyde dehydrogenase	8423281	LGG_RS10955	-0.489318713	0.043810793
Cell wall component (peptidoglycan) synthesis				
Glycosyltransferase family 4 protein	8421762	LGG_RS13715	-0.778576927	0.000250925
Glycosyltransferase family 2 protein	8422644	LGG_RS05060	-0.737444871	0.001439778
UTP--glucose-1-phosphate uridylyltransferase, GalU	8422528	LGG_RS05080	-0.681023608	0.002473912
PBP1A family penicillin-binding protein	8421232	LGG_RS08585	-0.582014575	0.010184645
Glycosyltransferase family 2 protein	8422114	LGG_RS01430	-0.536839936	0.023862409
**Cell wall synthesis inhibitor**				
Glycopeptide antibiotics resistance protein	8422194	LGG_RS12265	-0.579304335	0.016232524
Proteolysis				
PDZ domain-containing protein	8421728	LGG_RS13430	-1.270293101	1.24E-10
Adaptor protein MecA	8421235	LGG_RS08630	-0.802045492	0.000172854
Antioxidant Enzyme				
Peroxiredoxin	8422481	LGG_RS03425	-0.776587055	0.001237206
Protein stability				
Hsp20 alpha crystallin family protein	8421930	LGG_RS13420	-0.805388489	0.000695788
Carbohydrate metabolism				

Glycosyl hydrolase	8423362	LGG_RS03220	-0.722569546	0.002390032
Fructose-bisphosphatase class III	8421357	LGG_RS09780	-0.632724024	0.005994792
Mannose-6-phosphate isomerase, class I	8420606	LGG_RS01585	-0.542166939	0.023392107
Tagatose 1,6-diphosphate aldolase	8422757	LGG_RS12320	-0.479188277	0.042864249
**Ion transporters**				
OFA family MFS transporter	8423191	LGG_RS02840	-3.655396005	5.72E-73
ATPase	8421071	LGG_RS06320	-0.75394314	0.000462478
Cation-transporting P-type ATPase	8420650	LGG_RS02195	-0.587228231	0.008700047
Cell division and interaction inhibitors				
Peptidoglycan-binding protein LysM	8421318	LGG_RS09405	-1.09355648	1.43E-07
Septation ring formation regulator EzrA	8423204	LGG_RS06040	-0.553568163	0.016643675
DNA recombination				
Recombinase RecT	8420959	LGG_RS05280	-1.33856371	2.68E-08
ISNCY family transposase	8422907	LGG_RS14095	-0.590458249	0.00953064
Excinuclease ABC subunit UvrB	8420869	LGG_RS04415	-0.591938812	0.009798713
Signal transduction				
Response regulator transcription factor	8420902	LGG_RS04815	-0.821416561	0.000184681
HAMP domain-containing protein	8422948	LGG_RS04820	-0.768447297	0.000411862
HAMP domain-containing histidine kinase	8422604	LGG_RS08290	-0.54714459	0.019050217
Response regulator transcription factor	8422309	LGG_RS08295	-0.498033141	0.046008946
Uncharacterized				
Hypothetical protein	8421293	LGG_RS09180	-1.323343516	2.16E-11
Hypothetical protein	8420549	LGG_RS01025	-0.57785312	0.01004688
Hypothetical protein	8422375	LGG_RS12295	-0.815589173	0.000167638
Hypothetical protein	8423353	LGG_RS05950	-0.71116364	0.002106121
Hypothetical protein	8421168	LGG_RS07580	-0.569290293	0.021123349
Hypothetical protein	8421038	LGG_RS05955	-0.721545006	0.002562845
Hypothetical protein	8422778	LGG_RS05165	-1.274447454	1.06E-07
Hypothetical protein	8420942	LGG_RS05170	-0.830717159	0.000490516
DUF3800 domain-containing protein	8423169	LGG_RS02915	-0.576307587	0.011741486
DUF1002 domain-containing protein	8421636	LGG_RS12410	-0.630897815	0.005521314
Hemolysin III family protein	8422041	LGG_RS06700	-0.746420113	0.000476136
ssrA	31492720	LGG_RS14625	-0.598082611	0.006721748
PTS galactitol transporter subunit IIC	8422265	LGG_RS01640	-0.526898066	0.028543913
PTS sugar transporter subunit IIA	8422324	LGG_RS01650	-0.514457516	0.041581788
PTS sugar transporter subunit IIC	8420602	LGG_RS01530	-0.744435947	0.001974839
Iron-sulfur cluster biosynthesis family protein	8422318	LGG_RS13425	-1.265999633	1.75E-09
Iron-sulfur cluster biosynthesis family protein	8421314	LGG_RS09385	-0.884707649	5.70E-05
DegV family protein	8422290	LGG_RS06705	-0.471663664	0.048842406
GTPase HflX	8421337	LGG_RS09585	-0.594760524	0.010070172

DUF1542 domain-containing protein	8423347	LGG_RS08970	-0.686414782	0.001391541
VTT domain-containing protein	8420821	LGG_RS03810	-0.528596528	0.025470553
DUF975 family protein	8420587	LGG_RS01435	-0.485392102	0.047691096
