# Supplementary material for: Intestinal epithelial cell-derived components regulate transcriptome of Lactobacillus rhamnosus GG
Source: Front Microbiol. 2023 Jan 4;13:1051310. doi: 10.3389/fmicb.2022.1051310 (PMC9846326; doi:10.3389/fmicb.2022.1051310)
Supplement: Supplementary file 2 [file Data_Sheet_2.PDF]

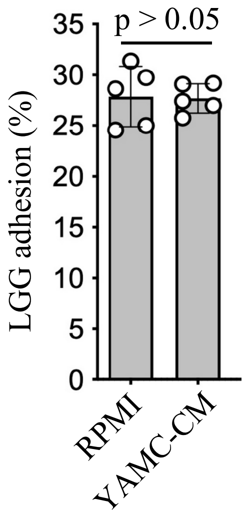

Supplementary Figure 1. Intestinal epithelial cell-derived components do not affect LGG adhesion to the mucin surface *in vitro*. YAMC-CM was prepared from 24-hour YAMC cell culture in RPMI. LGG was cultured in YAMC-CM or RPMI for 24 hours at 37ºC. Adhesion of LGG to the mucin surface was measured. % of LGG adhesion = (Adhere LGG /Initial loading LGG) x 100. Values are expressed as mean ± SEM. N=5 independent experiments.
