# Supplementary material for: Intestinal epithelial cell-derived components regulate transcriptome of Lactobacillus rhamnosus GG
Source: Front Microbiol. 2023 Jan 4;13:1051310. doi: 10.3389/fmicb.2022.1051310 (PMC9846326; doi:10.3389/fmicb.2022.1051310)
Supplement: Supplementary file 3 [file Data_Sheet_3.PDF]

Supplementary Materials and Methods

LGG adhesion assay

Mucin (partially purified porcine gastric mucin type III, Sigma-Aldrich) at 1 mg/ml in 10 mM HEPES/Hanks buffer was immobilized on 24 well polystyrene plate for 1 hour at room temperature and subsequently incubated overnight at 4°C. Then wells were washed twice with 10 mM HEPES/Hanks buffer. LGG was cultured with YAMC-CM or RPMI medium for 24 hours, washed with PBS, and suspended in PBS at 10^8 CFU/ml. LGG (3×10^7 CFU/well) was added to mucin-coated wells and incubated at 37 °C for 1 hour. Wells were thoroughly washed twice with PBS to remove unbound bacteria. 0.5% (v/v) Triton X-100 solution in PBS was added to the well and incubated at room temperature for 2 hours under gentle agitation to dislocate the bound bacteria. The number of LGG was examined by measurement of OD600 using a spectrophotometer. The percentage of adhesion was calculated as: (adhere LGG /total loading LGG) × 100.
